# Diffuse scattering in silver hypo­di­phos­phate, Ag_4_(P_2_O_6_), probed by 3D ED

**DOI:** 10.1107/S2053229626005012

**Published:** 2026-05-26

**Authors:** Vasyl Kinzhybalo, Jakub Wojciechowski, Dorota A. Kowalska, Vladyslav Maliuzhenko, Katarzyna A. Ślepokura

**Affiliations:** a Institute of Low Temperature and Structure Research Polish Academy of Sciences, 2 Okólna Wrocław 50-422 Poland; b Rigaku Europe SE, Hugenottenallee 167, Neu Isenburg, D-63263, Germany; c Faculty of Chemistry University of Wrocław, 14 F Joliot-Curie Wrocław 50-383 Poland; Rigaku Americas Corporation, USA

**Keywords:** powder diffraction, electron diffraction, silver hypo­di­phos­phate, diffuse scattering, real structure, geometric frustration

## Abstract

The crystal structure of silver hypo­di­phos­phate, Ag_4_(P_2_O_6_), was determined using 3D ED. The average structure is hexa­gonal, described in the space group *P*6_3_/*mcm*, and is isomorphous with the average structure of known Li_4_(P_2_S_6_). The silver cations form hexa­gonal atomic ring layers and the hypo­di­phos­phate anions occupy channels that centre every hexa­gonal ring, with the P—P bond oriented along the unique *c* axis. The hypo­di­phos­phate P_2_O_6_^4−^ anions are disordered, with the P atoms occupying two positions having 50% occupancy each and with O atoms common for both positions of the anion. The O atoms form an octa­hedral coordination environment for the silver cations. The hypo­di­phos­phate anions are stacked into columns along the unique axis direction. Neighbouring hypo­di­phos­phate columns may have P—P bonds on the same (ferro-type) or on different (anti­ferro-type) levels. This correlated disorder manifests itself in diffuse scattering observed on *hkl* layers with uneven *l* values. Simulations based on an Ising-type model with geometric frustration align well with the experimental data, providing insight into the short-range anti­ferro-like arrangement of disordered hypo­di­phos­phate anions.

## Abstract 2

A 3D ED study of silver hypo­di­phos­phate, Ag_4_(P_2_O_6_), has revealed diffuse scattering and was used to model real structure.

## Introduction

Nowadays, the crystal structure determination of new materials has become routine for even sub-micron monocrystalline samples, largely thanks to the state-of-the-art electron diffractometers (Ito *et al.*, 2021 ▸). Knowledge of the structure is important in terms of understanding the ‘com­position–structure–properties’ relationship, which is vital in the design of new materials with desired properties. However, sometimes the real structure differs from the average one, *i.e.* that determined by an anal­y­sis of Bragg diffraction. These differences may prove to be important and have a considerable effect on the properties of the material (Schmidt *et al.*, 2023 ▸). One of the well-known examples of such structures are the triangular lattices with anti­ferro-type inter­actions between closest neighbours (Keen & Goodwin, 2015 ▸). The inability to com­pletely satisfy the anti­ferro arrangement in the triangular lattice leads to disordered structures with eventual correlations in the disorder.

**Figure 1:**
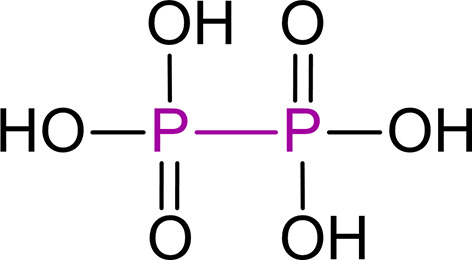
The structural formula of hypodi­phospho­ric acid.

Since the first report on hypodi­phospho­ric acid, H_4_P_2_O_6_ (Fig. 1 ▸), in 1877 by Theodor Salzer until nowadays, knowledge relating to the crystal structures of simple inorganic hypo­di­phos­phates remains quite limited, especially in com­parison with other common inorganic phos­phates (Kinzhybalo *et al.*, 2021 ▸). However, in recent years, there has been an increase in the number of reports of inorganic hypo­di­phos­phates: lithium, sodium and ammonium salts have been systematically investigated for their crystal structures and properties, including ionic conductivity in Na_4_(P_2_O_6_) (Szafranowska *et al.*, 2012 ▸; Kinzhybalo *et al.*, 2024 ▸; Ślepokura *et al.*, 2025 ▸; Otręba *et al.*, 2026 ▸), ferroelectricity in (NH_4_)_2_(H_2_P_2_O_6_) (Szklarz *et al.*, 2011 ▸), electron-density distribution in Li_4_(P_2_O_6_)·6H_2_O and bis­(guanidinium) disodium hepta­hydrate, (CH_6_N_3_)_2_Na_2_(P_2_O_6_)·7H_2_O (Kinzhybalo *et al.*, 2013 ▸; Starynowicz *et al.*, 2025 ▸), and thermal stability and spectroscopic properties of many inorganic salts have been reported (Gjikaj *et al.*, 2012 ▸; Wu *et al.*, 2012 ▸; Gjikaj & Wu, 2014 ▸; Gjikaj *et al.*, 2014 ▸; Wu *et al.*, 2015 ▸; Haase & Gjikaj, 2017 ▸; Haase & Gjikaj, 2018 ▸).

The first report on the synthesis of Ag_4_(P_2_O_6_) (using phospho­rus and a warm acidified solution of silver nitrate) dates back to 1883 (Philipp, 1883 ▸). It was also obtained by Joly in 1885 (Joly, 1885 ▸) and Salzer in 1886 (Salzer, 1877 ▸; Salzer, 1886 ▸) in double decom­position reactions. Silver hypo­di­phos­phate was used to obtain organic esters of hypodi­phospho­ric acid (Sänger, 1886 ▸). It was also utilized in a qu­anti­tative determination of hypo­di­phos­phate anions in the presence of phos­phate and phosphite (Wolf & Jung, 1931 ▸). Solid-state ^31^P NMR spectroscopy was reported for Ag_4_(P_2_O_6_) (Grimmer *et al.*, 1978 ▸). Its diffraction pattern along with the unit-cell parameters and space group were deposited in the Inter­national Centre for Diffraction Data (Kabekkodu & Blanton, 2024 ▸; Kabekkodu *et al.*, 2024 ▸) as PDF deposition number 00-047-0901 by H. Worzala in 1996, but a corresponding crystal structure has never been published in the literature. Accor­ding to the deposited data, Ag_4_(P_2_O_6_) crystallizes in the space group *P*6_3_/*mcm* and is isostructural with the Li_4_(P_2_S_6_) average structure (Mercier *et al.*, 1982 ▸). Both are characterized by disorder of the hypo­di­phos­phate anions within the ordered framework of metal cation positions. The structure of Li_4_(P_2_S_6_) is still under debate in the literature, as there are several reports on its proper space-group determination. The models solved in the space groups *P*
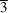
1*m*, *Pnnm* and *Pnma* were considered by Hood *et al.* (2016 ▸), but none was given a preference. Dietrich *et al.* (2016 ▸) used synchrotron PXRD and PDF anal­y­sis and favoured the model in the space group *P*
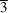
1*m*. Refinement using NMR crystallography was reported in the space group *P*321 (Neuberger *et al.*, 2018 ▸). The same non­centrosymmetric model was reported by Oxley *et al.* (2023 ▸) and confirmed by second and third harmonic generation measurements. Yahia *et al.* (2023 ▸) reported the twinned single-crystal structure solution in the space group *P*
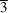
*m*1. The related selenium-containing material, Li_4_(P_2_Se_6_), turned out to crystallize as a similar, but not isomorphous, form to the thio-analogue crystal form (Neuberger *et al.*, 2025 ▸). The other related substance – effective sodium ionic conductor, sodium hexa­thio­hypo­di­phos­phate, Na_4_(P_2_S_6_) – is known to exist in three polymorphic modifications, but none of them is isomorphous with Li_4_(P_2_S_6_) (Scholz *et al.*, 2021 ▸; Scholz *et al.*, 2022 ▸). Similarly, the recently reported new ionic conductor Na_4_(P_2_O_6_) crystallizes in its own structure type, different from the above-mentioned substances (Kinzhybalo *et al.*, 2024 ▸). Knowledge of the proper crystal structure is crucial in un­der­standing lithium and sodium ion conductivity mechanisms in these materials (Li *et al.*, 2020 ▸; Stamminger *et al.*, 2020 ▸; Hogrefe *et al.*, 2025 ▸).

Considering the isomorphism of Li_4_(P_2_S_6_) and Ag_4_(P_2_O_6_), and the disorder of the anions in both com­pounds, it was inter­esting to perform detailed structural studies of the latter salt and record subtle diffraction effects that would allow the determination of its real crystal structure. Due to the limited solubility of silver hypo­di­phos­phate in water, crystallization from aqueous solution gives a nanocrystalline material. Therefore, electron diffraction was employed to determine the crystal structure of Ag_4_(P_2_O_6_), and to record and study the diffuse scattering that originates from the correlations in disorder of the hypo­di­phos­phate anions.

## Experimental

The obtained material was characterized using scanning electron microscopy (SEM) with energy dispersive X-ray anal­y­sis (EDX), three-dimensional electron diffraction (3D ED), powder X-ray diffraction (PXRD), variable-tem­per­a­ture PXRD (VT-PXRD), thermogravimetry–differential scanning calorimetry (TG–DSC) and variable-tem­per­a­ture optical microscopy.

### Preparation

Hypodi­phospho­ric acid was obtained by red phospho­rus oxidation with H_2_O_2_ and was further neutralized with NaOH to give crystalline Na_2_(H_2_P_2_O_6_)·6H_2_O (Yoza & Ohashi, 1965 ▸). The title material was obtained by mixing stoichiometric amounts of Na_2_(H_2_P_2_O_6_)·6H_2_O (314 mg, 1.00 mmol) and Ag_2_SO_4_ (624 mg, 2.00 mmol) dissolved in a minimum amount of distilled water. A white precipitate was formed immediately and gradually darkened over time. The precipitate was filtered off, washed with water and dried, yielding a slightly brownish grey powder.

### Methods

#### Scanning electron microscopy (SEM) with energy dispersive X-ray anal­y­sis (EDX)

The electron microscopy imaging of the sample and its ele­men­tal com­position were studied using the field-emission scanning electron microscope (FE-SEM) FEI Nova NanoSEM 230, along with an energy dispersive X-ray spectrometer (EDAX Genesis XM4).

#### Thermogravimetry–differential scanning calorimetry (TG–DSC)

TG–DSC anal­y­sis of Ag_4_(P_2_O_6_) (12.57 mg) was performed using a Mettler–Toledo TGA/DSC 3+ instrument in the tem­per­a­ture range 303–1073 K with a ramp rate of 10 K min^−1^. The scans were performed in flowing nitro­gen (flow rate: 3 dm^3^ h^−1^).

#### Variable-tem­per­a­ture microscopy

Optical observations were carried out on an Olympus BX53 microscope equipped with a Linkam THMS 600 tem­per­a­ture adapter and a CCD XC50 video camera in the tem­per­a­ture range 293–593 K.

#### Room-tem­per­a­ture and variable-tem­per­a­ture PXRD

The PXRD data for the Rietveld refinement of Ag_4_(P_2_O_6_) were collected at room tem­per­a­ture on a PANalytical X’Pert Pro θ–2θ powder X-ray diffractometer using β-filtered Cu *K*α radiation in the 2θ range 15–132°, with a scan step of 0.007°. The background was fitted as a 9-parameter polynomial. Peak shape was approximated with a pseudo-Voigt profile function. A scale factor and specimen displacement were refined. The coordinates (with special position restrains) and anisotropic *B* factors of all the atoms were refined.

Variable-tem­per­a­ture powder X-ray diffraction (VT-PXRD) anal­y­sis of the Ag_4_(P_2_O_6_) sample was performed on the same PANalytical X’Pert Pro diffractometer with an Anton Paar HTK 1200N high-tem­per­a­ture chamber. Data were collected in the 2θ range 10–90° every 50 K in the tem­per­a­ture range 300–850 K. All Rietveld refinements were performed using the *HighScore Plus* program (Degen *et al.*, 2014 ▸).

#### Electron diffraction data collection and refinement

The crystal structure was determined by 3D electron dif­fraction (3D ED) from a *ca* 400 nm single crystal rotated about one axis by 120° (scan width 0.5°, 240 images) and with a 0.5 s/° exposure time. The data were collected at ambient tem­per­a­ture *in vacuo* using a Rigaku Synergy-ED dif­frac­tom­eter equipped with a Rigaku HyPix-ED detector optimized for electron detection and an LaB_6_ electron source at 200 kV (λ = 0.0251 Å) (Ito *et al.*, 2021 ▸). Data collections, cell refinements, data reductions and anal­y­sis were carried out with *CrysAlis PRO* (Rigaku OD, 2022 ▸). Due to the high symmetry of the phase, this single scan resulted in 87% com­pleteness (up to 0.6 Å resolution). The structure was solved with *SHELXT* (Sheldrick, 2015 ▸). Both kinematical and dynamical refinements were carried out in *OLEX2* (Dolomanov *et al.*, 2009 ▸). Anisotropic displacement parameters for all atoms were refined. The electron scattering atomic factors of UCLA were used (Saha *et al.*, 2022 ▸). The average crystal structure model from dynamical 3D ED refinement (hexa­gonal crystal system, space group *P*6_3_/*mcm*) was finally refined *versus* powder diffraction data.

Details of the kinematical and dynamical crystal structure refinements, along with the PXRD Rietveld fit details, are given in Table 1 ▸.

The *DIAMOND* program (Brandenburg, 2022 ▸) was used to produce the figures. All diffraction patterns in this study were calculated using the program *DIFFUSE* (Proffen & Neder, 1997 ▸).

## Results and discussion

### Sample characterization

**Figure 2:**
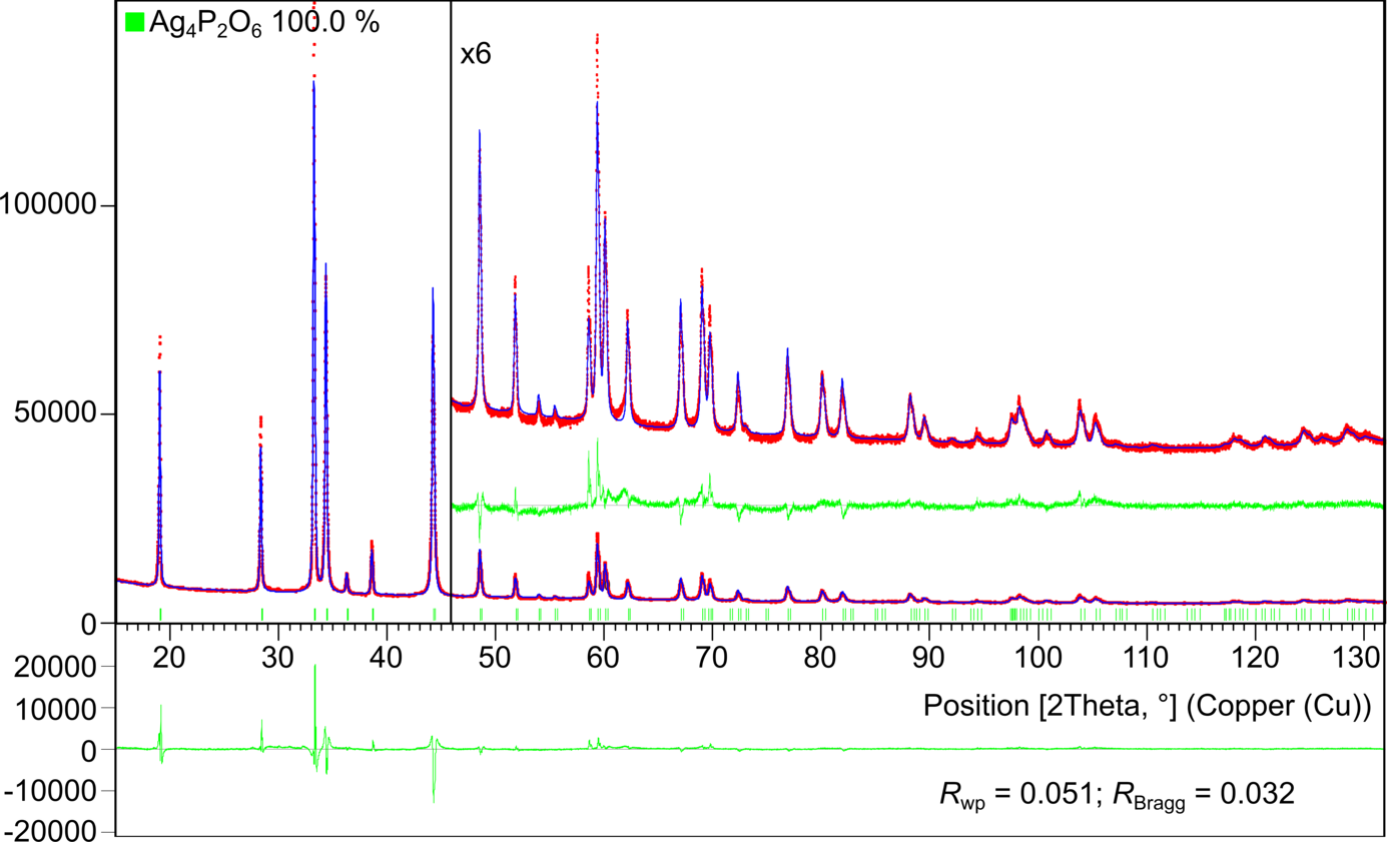
Rietveld fit of the PXRD data of the bulk sample using the average structure model from the dynamical refinement of the 3D ED data (room tem­per­a­ture, 2θ range 15–132°).

As mentioned above, the diffraction pattern, unit-cell parameters and space group, but not the atomic coordinates, for Ag_4_(P_2_O_6_) were revealed in the database (ICDD, PDF deposition number 00-047-0901) (Kabekkodu & Blanton, 2024 ▸; Kabekkodu *et al.*, 2024 ▸). We determined the crystal structure of the title com­pound by 3D ED and used the atomic model for the Rietveld refinement against the powder diffraction data (Fig. 2 ▸ and Fig. S1 in the supporting information). PXRD showed no signs of crystalline silver or any other impurities.

**Figure 3:**
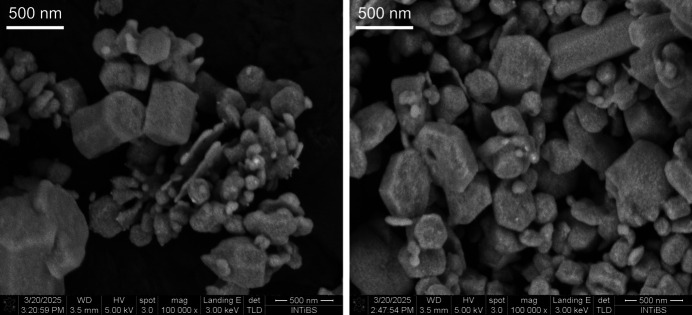
Representative electron microscopy images of the bulk sample (*cf* Figs. S2 and S3 in the supporting information).

SEM images with EDX anal­y­sis of the sample of the title com­pound showed hexa­gonal plate- and rod-shaped crystals, and confirmed the Ag_4_(P_2_O_6_) com­position (Fig. 3 ▸ and Figs. S2 and S3 in the supporting information).

### Stability

**Figure 4:**
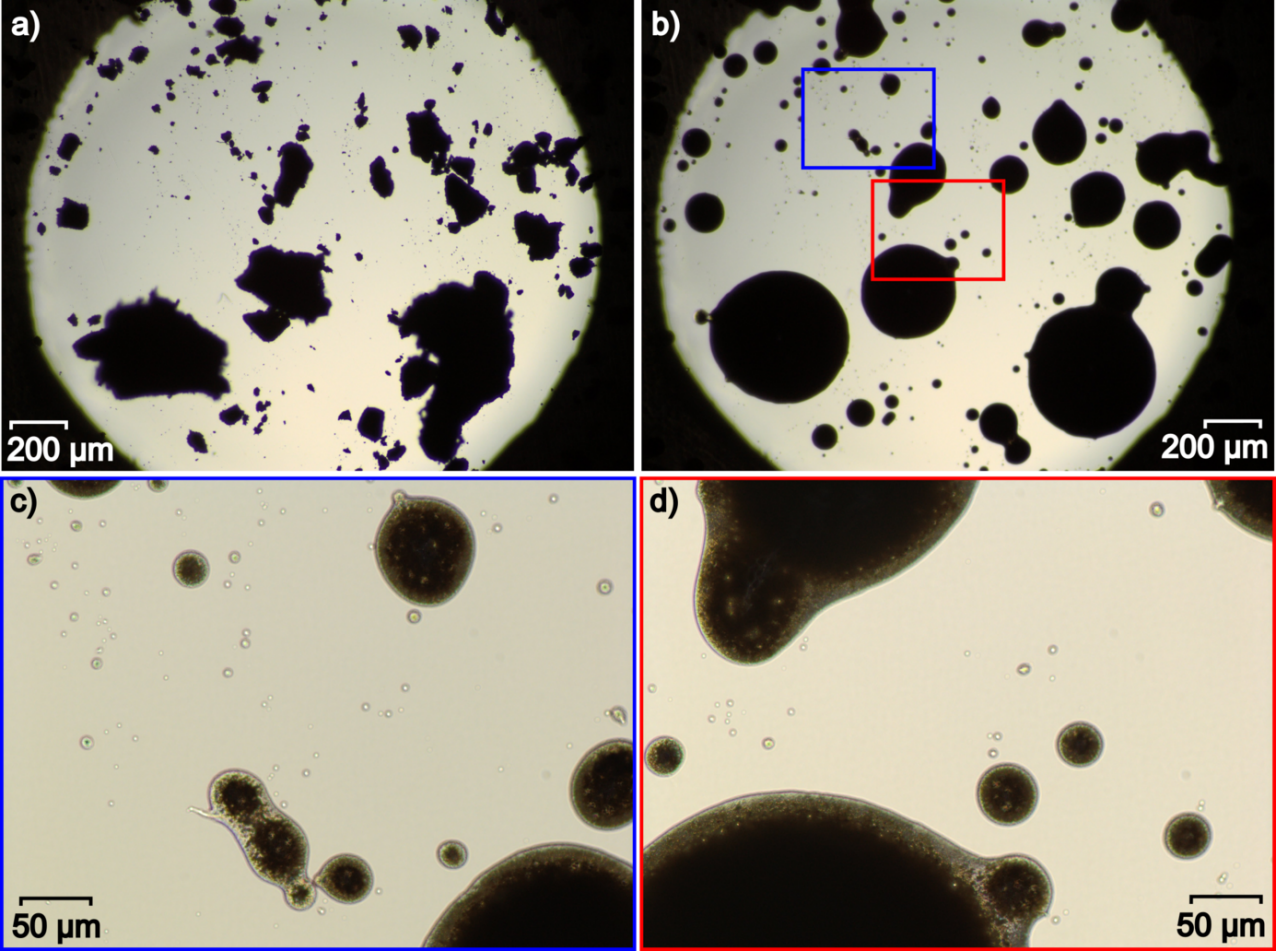
(*a*) Ag_4_(P_2_O_6_) powder before heating from 293 to 593 K, (*b*) after cooling from 593 to 293 K, along with zoomed (*c*) blue and (*d*) red areas (*cf* Fig. S5 in the supporting information).

Silver hypo­di­phos­phate undergoes partial decom­position within a few minutes after its precipitation, and the snowy white material turns greyish. Despite that, powder diffraction of several months old samples does not reveal any reflections from additional crystalline phases. Thermogravimetric anal­y­sis combined with differential scanning calorimetry (TG–DSC, sample mass *m* = 12.57 mg, ramp rate = 10 K min^−1^) does not reveal any considerable mass loss (Δ*m* = 6.7 × 10^−2^ mg, 0.5%) in the whole tem­per­a­ture range from 303 to 1073 K. However, the DSC curve shows several energetic processes in this range: three exothermic, around 500, 620 and 1020 K, and one endothermic, around 750 K (Fig. S4 in the supporting information). Variable-tem­per­a­ture optical microscopy performed in the tem­per­a­ture range 293–593 K (40 K min^−1^), along with variable-tem­per­a­ture powder diffraction (VT-PXRD), were used for a better description and documentation of the processes. As seen in Fig. 4 ▸ (and Fig. S5 in the supporting information), the powder melts between 533 and 543 K, and transforms into semi-transparent glassy drops.

**Figure 5:**
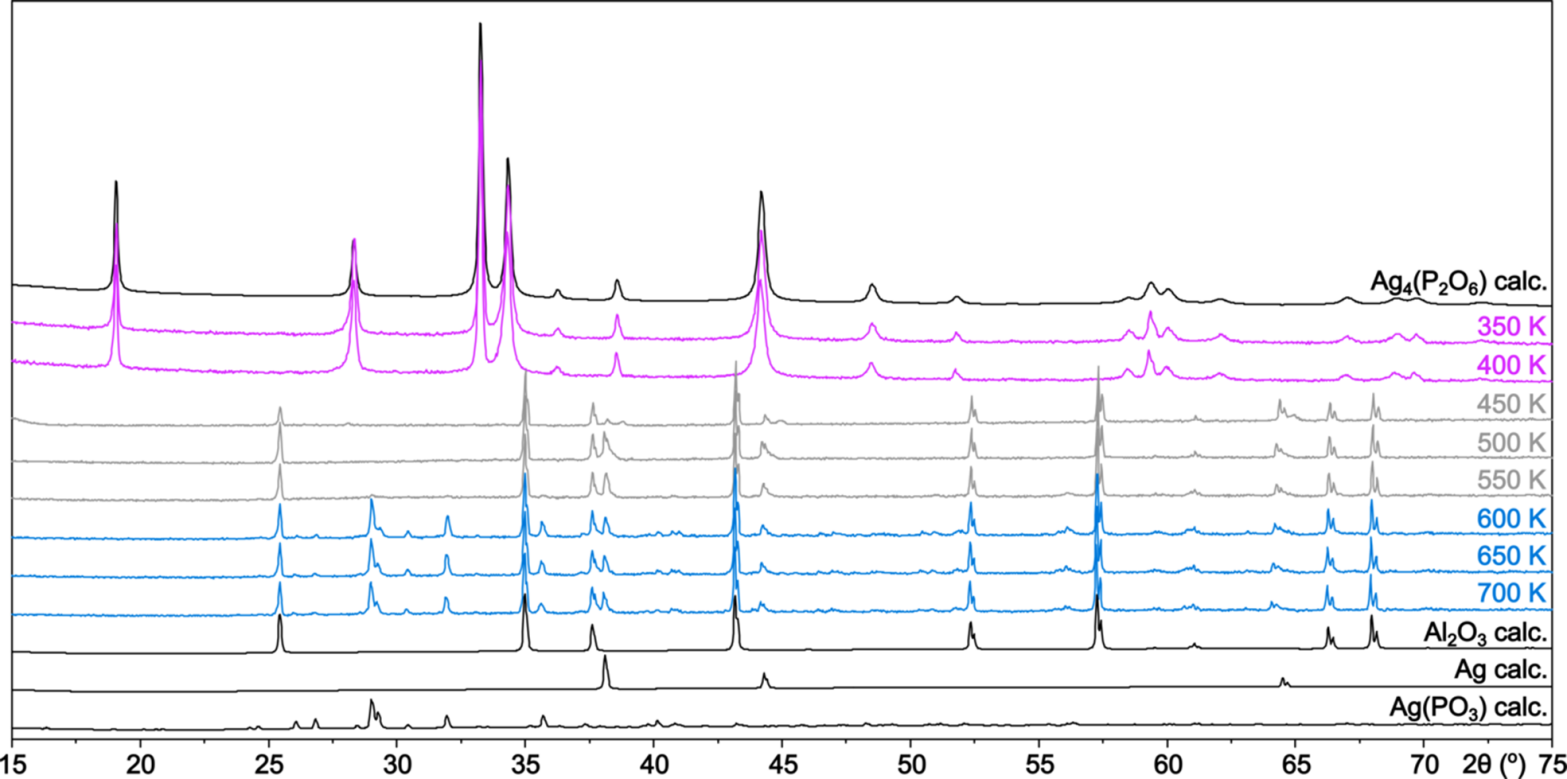
The variable-tem­per­a­ture powder X-ray diffraction (VT-PXRD) patterns for the sample of Ag_4_(P_2_O_6_) (2θ range 15–75°, recorded on heating every 50 K in the tem­per­a­ture range 350–700 K, shown from top to bottom). Calculated diffractograms are shown in black and experimental diffractograms are shown in violet, grey and blue.

VT-PXRD experiments, collected every 50 K between 300 and 850 K, have confirmed that Ag_4_(P_2_O_6_) is stable up to about 400 K (violet diffractograms in Fig. 5 ▸). At 450 K, decom­position begins and reflections from Ag appear (due to melting/decom­position and aggregation of the sample, the diffraction lines from the corundum sample holder also become visible; grey diffractograms in Fig. 5 ▸). On further heating, the intensities of the diffraction lines from metallic silver increase, and at 600 K, reflections from Ag(PO_3_) appear (blue diffractograms in Fig. 5 ▸) (Terebilenko *et al.*, 2011 ▸). This observation is consistent with the exothermic nature of the second peak on the DCS curve at about 620 K (crystallization). The crystalline Ag(PO_3_) formed in this way is stable up to a tem­per­a­ture of 700 K. Then, at about 750 K, it melts (literature m.p. 755–761 K; Osterheld & Mozer, 1973 ▸), which is accom­panied by the disappearance of the diffraction pattern in PXRD and the endothermic anomaly in the DSC curve at 750 K. The decom­position takes place as a redox process according to the equation:

Ag_4_(P_2_O_6_) → 2Ag(PO_3_) + 2Ag,

and is consistent with the observations of Philipp (1883 ▸).

### Average crystal structure

The average structure is the time- and space-averaged model derived from diffraction (Rietveld refinement), pro­viding a perfect periodic symmetry that lacks local detail. Real structure refers to the instantaneous, accurate and local atomic positions (obtained from PDF, EXAFS, diffuse scattering), capturing all disorder, defects and thermal vibrations.

The crystal structure of silver hypo­di­phos­phate, Ag_4_(P_2_O_6_), was determined using 3D ED. The average structural model obtained in this way was used for the Rietveld refinement of the PXRD data.

**Figure 6:**
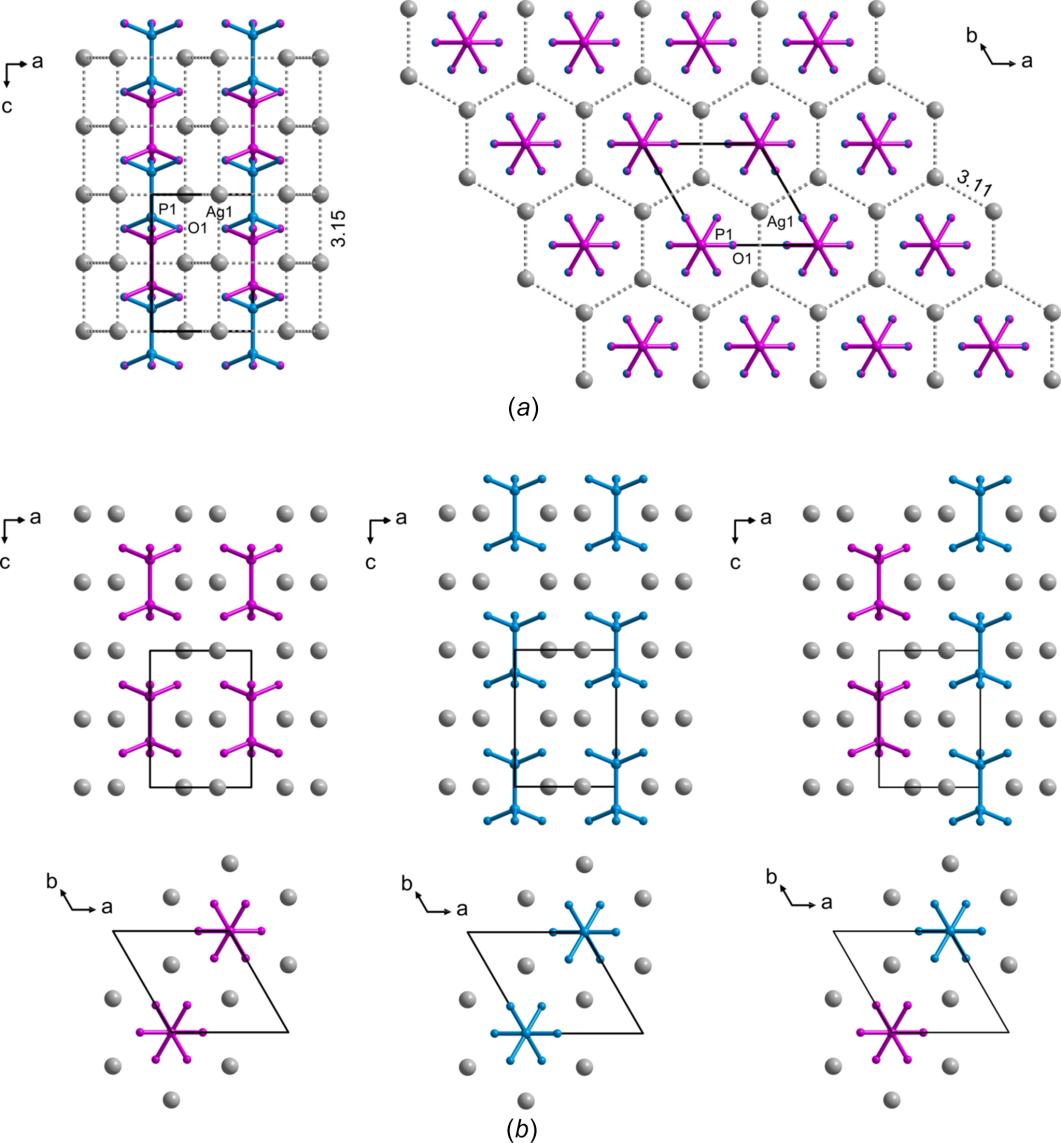
(*a*) Crystal structure packing views of Ag_4_(P_2_O_6_) along with (*b*) possible different mutual arrangements of the hypo­di­phos­phate anions shown in violet and blue colours. The Ag⋯Ag distances in part (*a*) are given in Å.

The average structure is hexa­gonal, described by *P*6_3_/*mcm* space-group symmetry, and isomorphous with the average structure of Li_4_(P_2_S_6_) (Mercier *et al.*, 1982 ▸). It should be noted that both known polymorphic modifications of Ag_4_(P_2_S_6_) crystallize as non-isomorphous with the Ag_4_(P_2_O_6_) structure types (Toffoli *et al.*, 1982 ▸; Toffoli *et al.*, 1983 ▸). The silver cations in Ag_4_(P_2_O_6_) form a three-dimensional substructure, in which hexa­gonal atomic ring ‘layers’ (perpendicular to [001]) can be distinguished. The ‘layers’ are arranged one above the other, forming channels along the *c*-axis direction. The metal centres are in close contact (Ag⋯Ag distances within the layers are 3.11 Å and Ag⋯Ag distances between the layers = 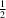
*c* = 3.15 Å), which indicates argentophilic inter­actions (Schmidbaur & Schier, 2015 ▸). Hypodi­phos­phate anions, P_2_O_6_^4−^, occupy the channels, with the P—P bond oriented along the the unique *c* axis. P atoms are disordered into two positions, each of which is 50% occupied, which means that the whole hypo­di­phos­phate anion occupies two positions [shown as violet and blue in Fig. 6 ▸(*a*)]. O-atom positions are common to both disorder com­ponents and create an octa­hedral coordination environment for the silver cations (Fig. S6). Hypodi­phos­phate anions are stacked into columns along the *c*-axis direction. Each column is chemically and structurally identical, but neighbouring columns may have P—P bonds on the same or on different levels [com­pare with the example arrangements of adjacent columns shown in Fig. 6 ▸(*b*)].

### Diffuse scattering

**Figure 7:**
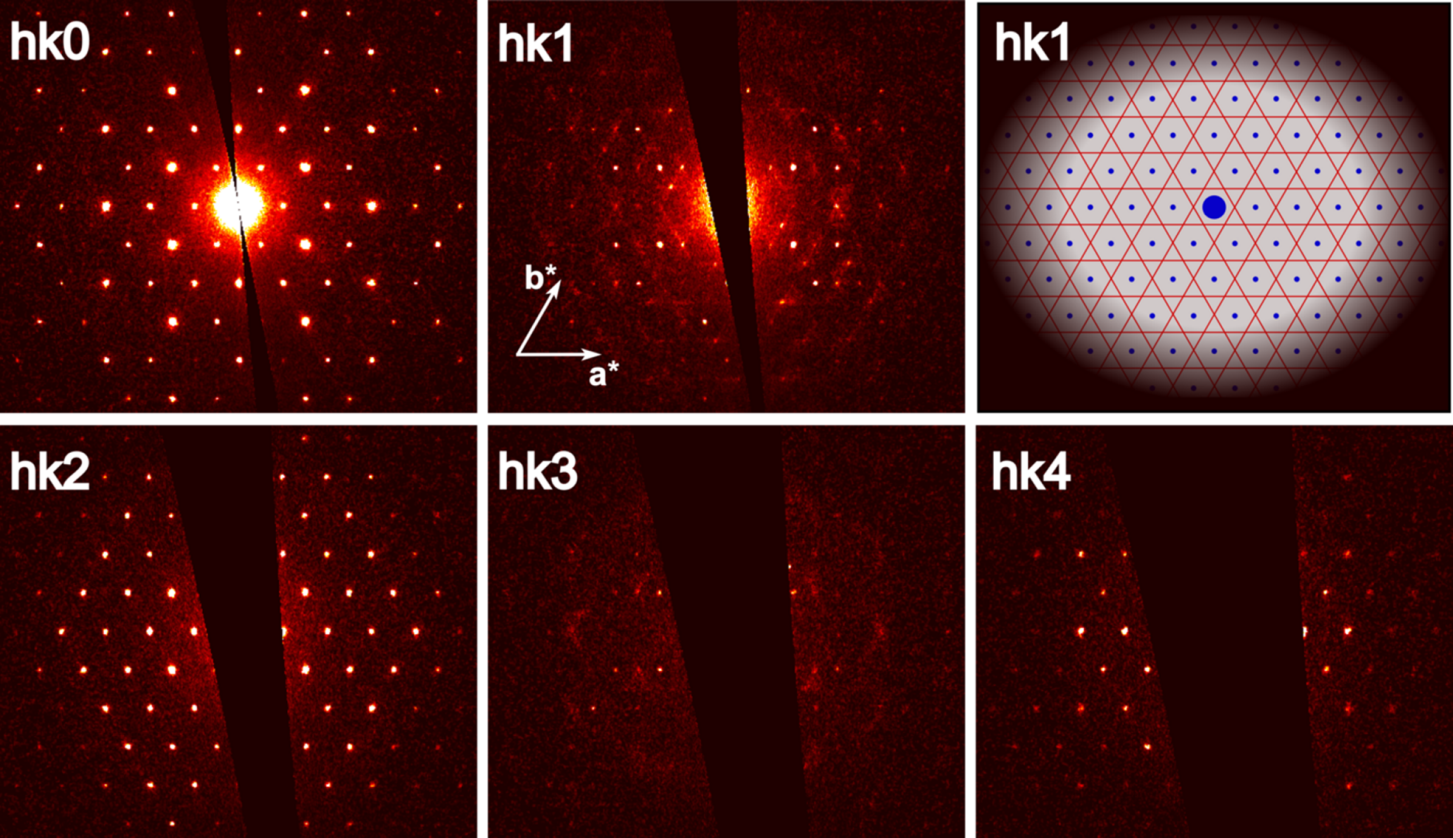
Ewald sphere reconstructions with diffuse scattering seen on *hkl* layers with *l* = 1 and *l* = 3, along with the schematic view of the diffuse streaks shown as red lines, Bragg reflections as small blue dots and the primary beam as a large blue dot (*cf* Fig. S7 in the supporting information).

The observed diffuse scattering (DS) lines on the *hkl* reciprocal planes shown in Fig. 7 ▸ (where *l* is odd, *l* = 2*m* + 1) appear midway between Bragg reflections along the a*, b* and a*–b* directions. This suggests short-range order in the crystal, with local doubling of the unit-cell parameters along the [100], [010] and [110] directions. The origin of this effect is in the occupational disorder at the P-site, which is half-occupied. However, along the *c*-axis direction, the P atoms are perfectly ordered due to the nature of the structure, resulting in the long-range order along this axis.

In contrast, along the [100], [010] and [110] directions, the atomic arrangement allows for multiple configurations, but still tends toward a local ordering. This local ordering forms ordered layers perpendicular to these three directions, and the diffuse scattering lines observed between Bragg peaks are a direct consequence of these short-range atomic correlations. The overall structure retains long-range order in the *c*-axis direction, but exhibits short-range order along the other directions, providing insight into the atomic arrangements in the disordered regions.

To propose a model of short-range order, it is essential to consider the concept of ‘geometric frustration’. This well-established idea has been applied previously to explain diffuse scattering effects in various crystal systems (Welberry *et al.*, 2011 ▸). We adopted a similar approach to investigate the spatial distribution of two distinct types of scatterers located on the triangular lattice of the *ab* plane in a hexa­gonal crystal. In our system, two possible anion configurations exist – designated as **A** and **B** (repre­sent­ed in blue and violet in Fig. 6 ▸). When an alternating arrangement of **A** and **B** is energetically favourable, such ordering is readily achievable on a square lattice but inherently frustrated on a triangular one. Specifically, when two sides of a triangle are occupied by alternating anions (**A** and **B**), the third must necessarily result in a like-pair (**AA** or **BB**), making perfect alternation geometrically impossible.

To implement such a model, Monte Carlo (MC) simulations based on the Ising model were employed. This approach has been used successfully in numerous studies to model various types of disorder in crystals (Welberry, 2004 ▸; Welberry *et al.*, 2011 ▸; Komornicka *et al.*, 2014 ▸; Bednarchuk *et al.*, 2017 ▸; Kowalska *et al.*, 2021 ▸). Simulations were performed for a series of cases using correlation parameters for both nearest-neighbour (*J*_1_) and next-nearest-neighbour (*J*_2_) inter­actions. The correlations were defined such that when *J_i_* < 0, neighbouring sites tend to differ in type.

**Figure 8:**
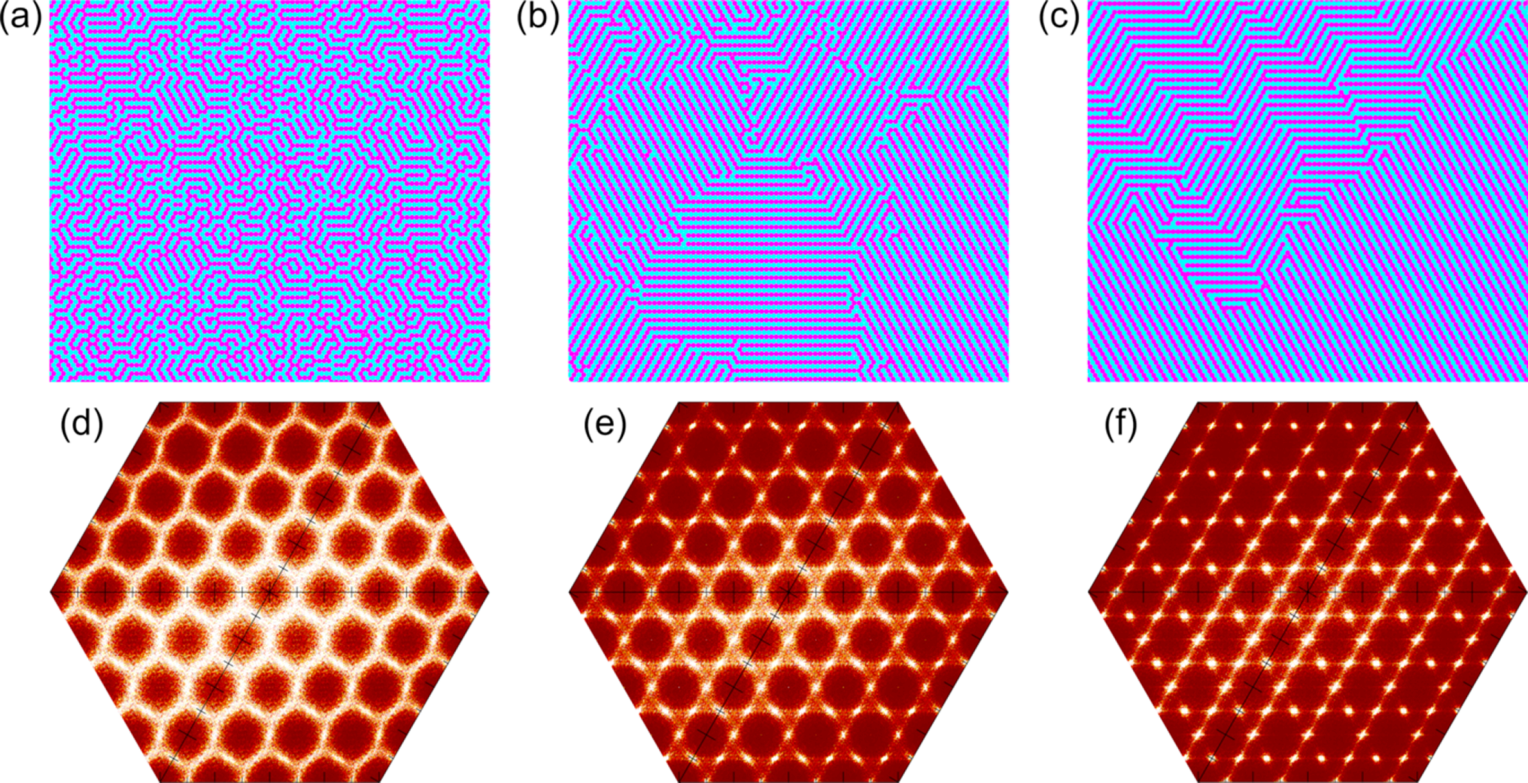
Example realizations of the Ising model with *J*_1_ = −5.0 and varying values of *J*_2_: (*a*) *J*_2_ = 0.0, (*b*) *J*_2_ = −0.3 and (*c*) *J*_2_ = −3.0. Corresponding calculated *hk*1 sections of reciprocal space are shown in parts (*d*)–(*f*), respectively.

The calculated models consist only of P ions, as the remaining atoms in the structure are fully ordered and do not contribute to the observed diffuse scattering effects. Three examples are pre­sent­ed in Fig. 8 ▸. In these simulations, the value of *J*_1_ was fixed at −5.0, while *J*_2_ was varied. Figs. 8 ▸(*a*)–(*c*) show the lattice configurations corresponding to each model, providing an overview of the spatial arrangements. The associated diffraction patterns (*hk*1 section) calculated from these configurations, are shown in Figs. 8 ▸(*d*)–(*f*).

In Fig. 8 ▸(*d*), with *J*_2_ = 0, the diffuse scattering appears as featureless diffuse rings. Introducing a small negative value for *J*_2_ (−0.3) leads to narrowing of the diffuse features and the appearance of distinct intensity enhancements midway between pairs of Bragg peaks [Fig. 8 ▸(*e*)]. Further decreasing *J*_2_ to −3.0 results in the weakening of diffuse streaks and a marked increase in additional intensity maxima [Fig. 8 ▸(*f*)]. Fig. 8 ▸(*e*) shows characteristics very similar to the observed patterns; therefore, the distribution in Fig. 8 ▸(*b*) can be considered the most representative of the short-range order present in the studied nanocrystal. Ewald sphere reconstructions based on the selected model, corresponding to those pre­sent­ed in Fig. 7 ▸, are shown in Fig. S8 in the supporting information.

## Conclusions

The average crystal structure of silver hypo­di­phos­phate, Ag_4_(P_2_O_6_), was determined from 3D electron diffraction and Rietveld fitting against PXRD data. The hexa­gonal structure is characterized by the statistical disorder of hypo­di­phos­phate anions, perfectly correlated along a unique axis direction and geometrically frustrated in the *ab* plane. Electron diffraction data revealed diffuse scattering between Bragg peaks on *hkl* layers with odd values of *l*, that originates from the presence of locally correlated anion arrangements, incom­patible with long-range periodicity. Simulations using a frustrated Ising model reproduce the key features of the experimental data, providing insight into the nature of short-range order in the crystal.

## Supplementary Material

Crystal structure: contains datablock(s) exp_7626_kinem, exp_7626_dynam, Ag4P2O6_5-140_17h_p_01, global. DOI: 10.1107/S2053229626005012/eq3025sup1.cif

Structure factors: contains datablock(s) exp_7626_kinem. DOI: 10.1107/S2053229626005012/eq3025exp_7626_kinemsup3.hkl

Structure factors: contains datablock(s) exp_7626_dynam. DOI: 10.1107/S2053229626005012/eq3025exp_7626_dynamsup2.hkl

Rietveld powder data: contains datablock(s) Ag4P2O6_5-140_17h_p_01. DOI: 10.1107/S2053229626005012/eq3025Ag4P2O6_5-140_17h_p_01sup4.rtv

Additional figures. DOI: 10.1107/S2053229626005012/eq3025sup5.pdf

CCDC references: 2553762, 2553761, 2553760

## Figures and Tables

**Table 1:** Crystallographic data for kinematical and dynamical refinement *versus* ED data, and Rietveld refinement *versus* PXRD data

Chemical formula	Ag_4_P_2_O_6_
*M*_r_ (g mol^−1^)	589.42
Crystal system, space group	Hexagonal, *P*6_3_/*mcm*
Temperature (K)	293
*a*, *c* (Å)	5.39128 (7), 6.30229 (9)
*V* (Å^3^)	158.640 (5)
*Z*	1
	
3D ED refinement	
Radiation type	200 kV electron beam (λ = 0.0251 Å)
θ range (°)	0.153–1.162
*R*_1_ (kinematical/dynamical refinement)	0.116/0.122
*wR*_2_ (kinematical/dynamical refinement)	0.400/0.281
	
Rietveld refinement	
Radiation type	Cu *K*α (λ = 1.5418 Å)
*R* (Bragg)	0.032
*R* (expected)	0.012
*R* (profile)	0.034
*R* (weighted profile)	0.051

## Data Availability

The published raw data, along with any supporting information not included in the article, is available from the authors upon reasonable request.
