# Supplementary material for: Diffuse scattering in silver hypo­di­phos­phate, Ag4(P2O6), probed by 3D ED
Source: Acta Crystallogr C Struct Chem. 2026 May 26;82(Pt 6):277–84. doi: 10.1107/S2053229626005012 (PMC13237487; doi:10.1107/S2053229626005012)
Supplement: Supplementary file 5 [file c-82-00277-sup5.pdf]

STRUCTURAL CHEMISTRY

Volume 82 (2026)

Supporting information for article:

Diffuse scattering in silver hypodiphosphate, $Ag_4(P_2O_6)$, probed by 3D ED

Vasyl Kinzhybalo, Jakub Wojciechowski, Dorota A. Kowalska, Vladyslav Maliuzhenko and Katarzyna A. Ślepokura

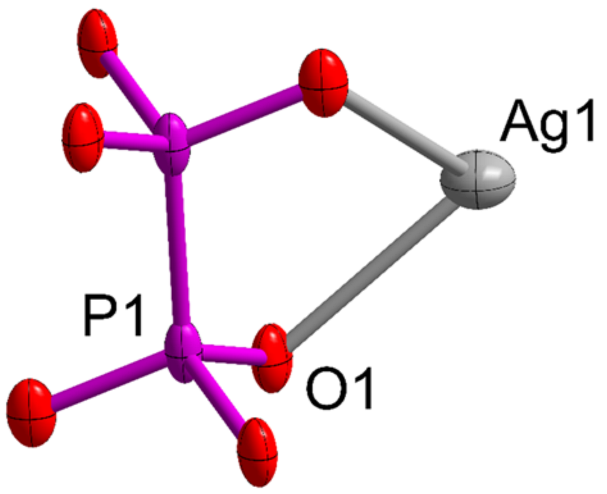

Figure S1 Structural unit of the title compound with the atoms of the asymmetric part labelled.

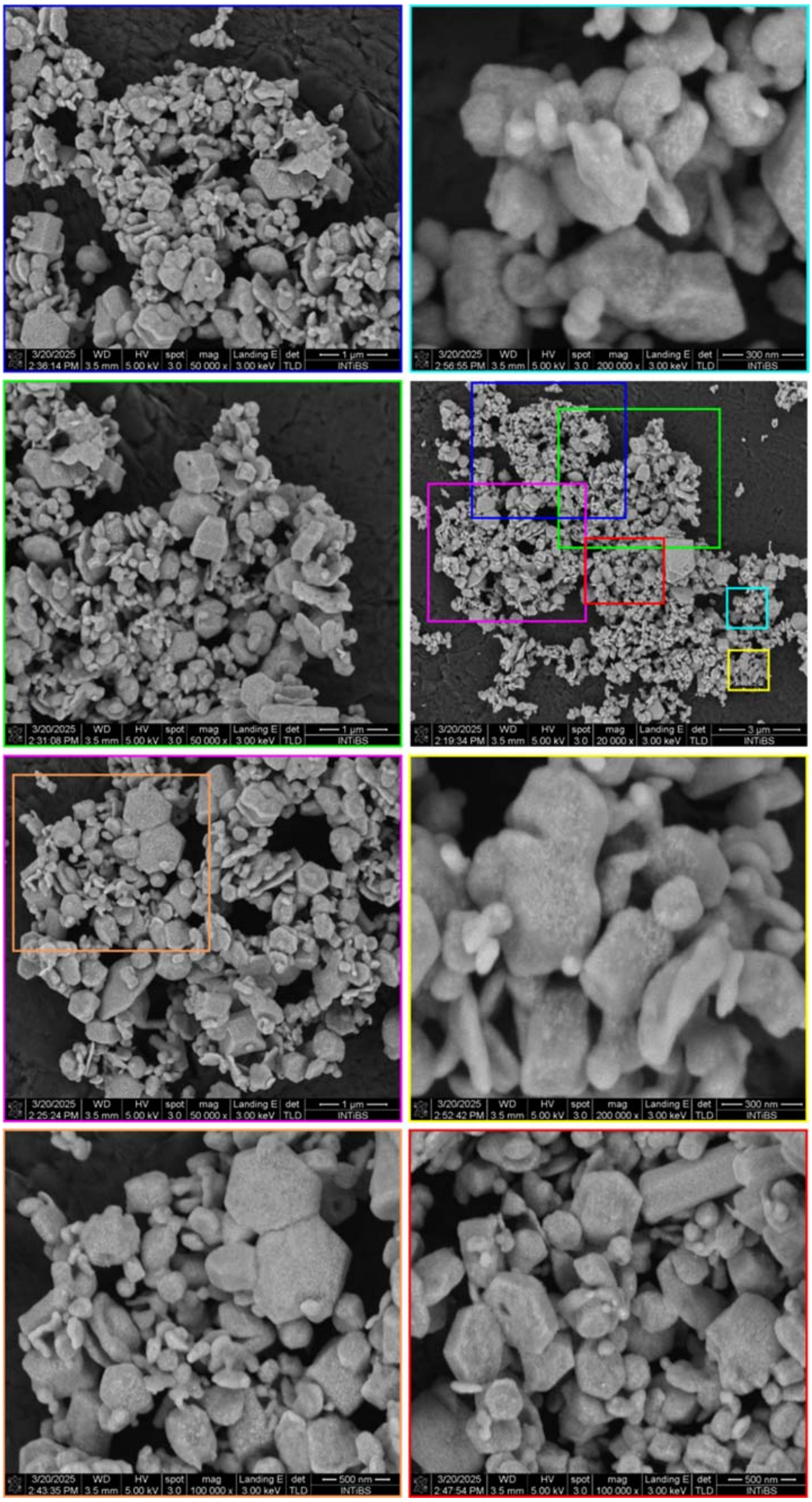

Figure S2 SEM images of $Ag_4(P_2O_6)$.

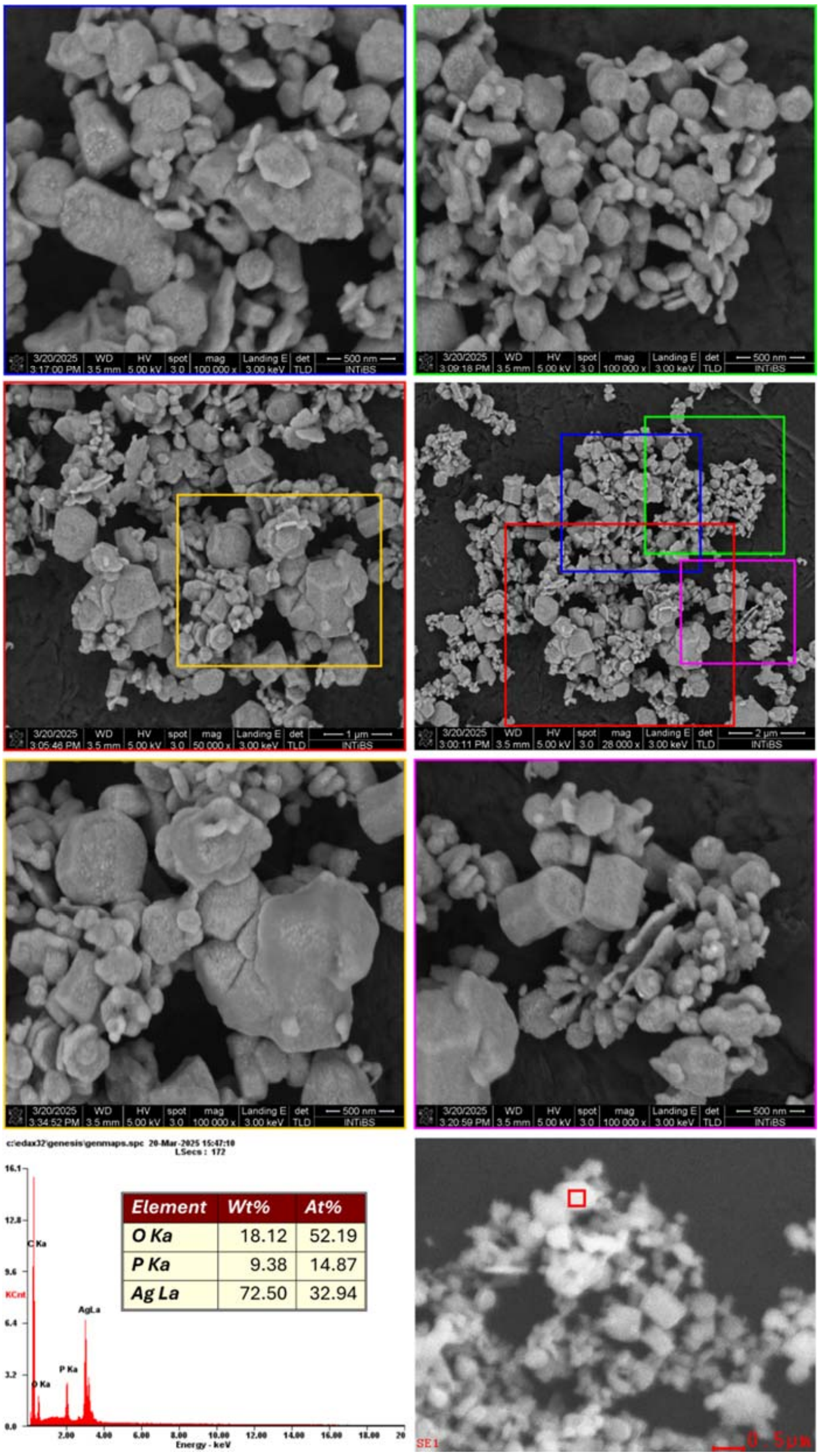

Figure S3 SEM images (top) and EDX analysis (bottom) of $Ag_4(P_2O_6)$. Theoretical content by mass: Ag 73.2%, P 10.51% and O 16.29%.

S1. Thermal stability

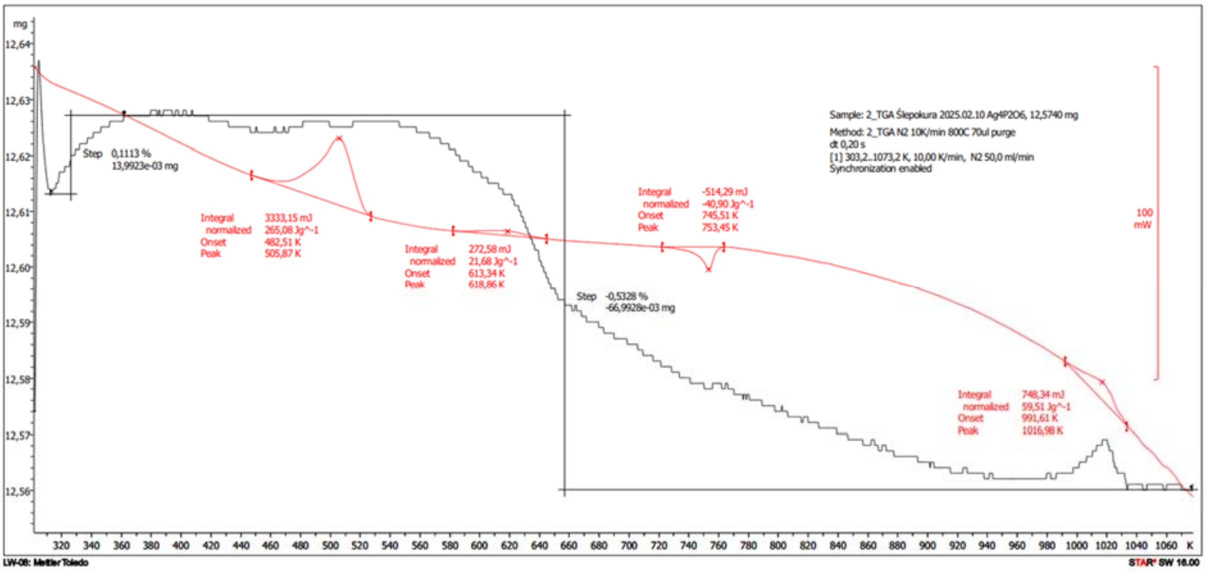

Figure S4 TG-DSC analysis of $Ag_4(P_2O_6)$. *m* = 12.57 mg. Practically no mass loss observed in the studied temperature range. Exo ↑.

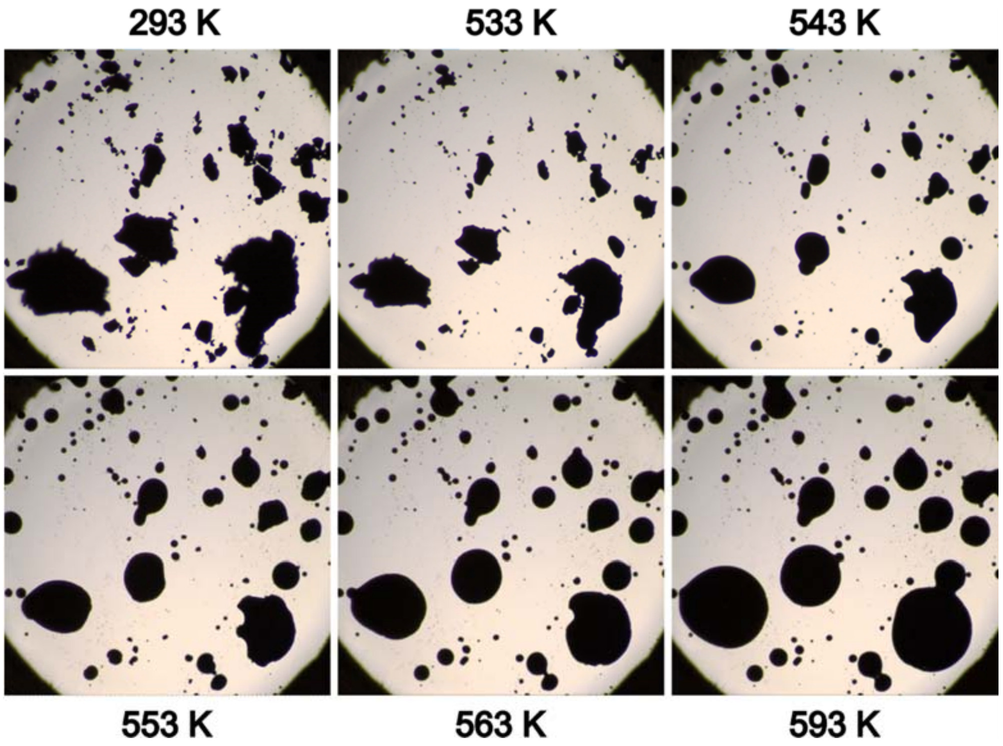

Figure S5 $Ag_4(P_2O_6)$ powder when heated from 293 to 593 K.

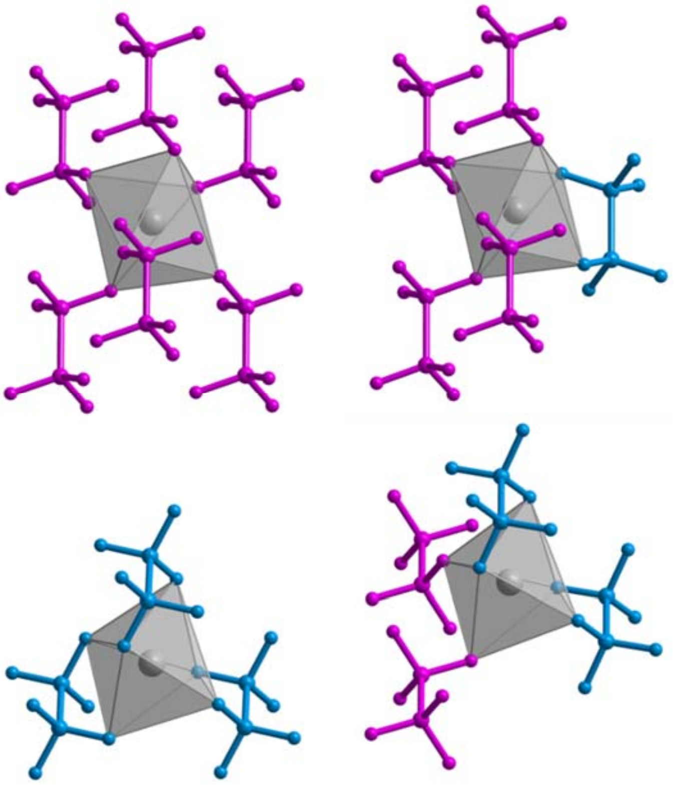

Figure S6 Possible coordination environments of the Ag^{+} cation (drawn in grey) in $Ag_4(P_2O_6)$.

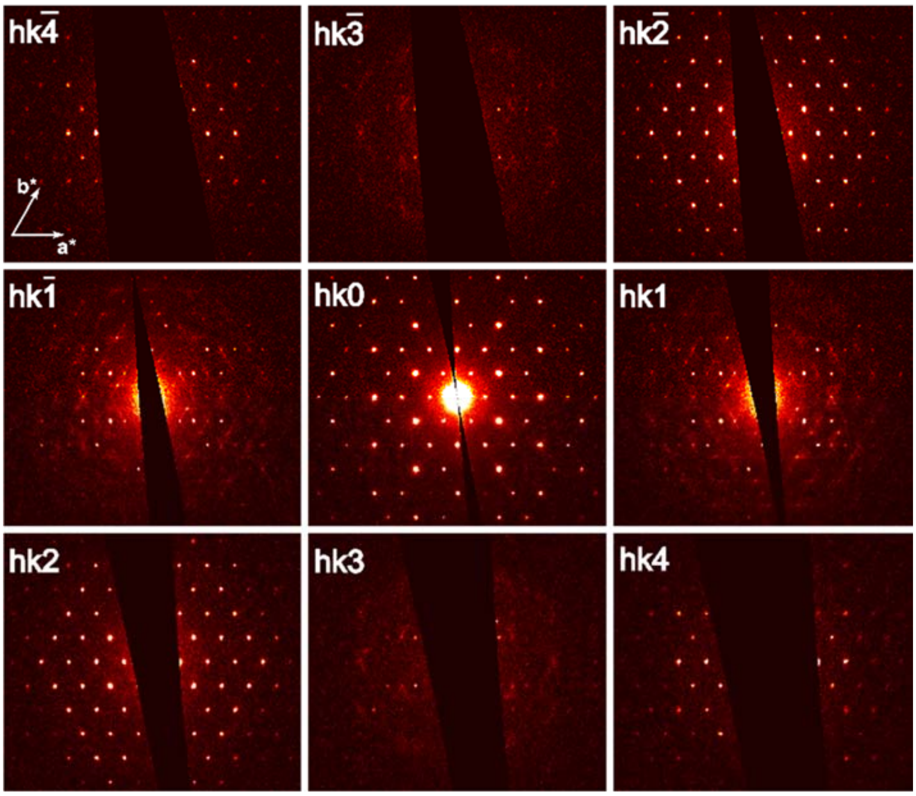

Figure S7 Ewald sphere reconstructions with diffuse scattering seen on *hkl* layers with uneven *l*.

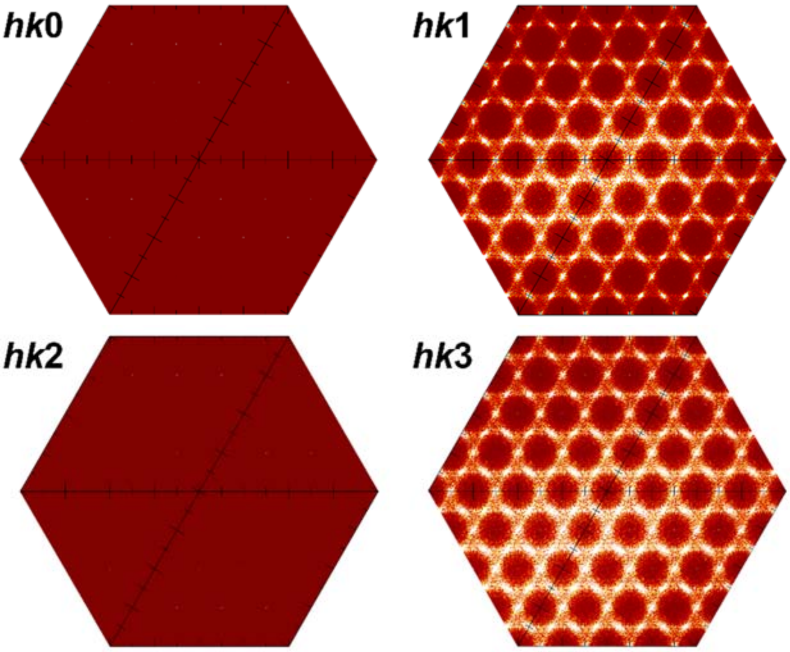

Figure S8 Reciprocal space sections of the model with correlation parameters $J_1 = –5.0$ and $J_2 = –0.3$. Diffuse scattering effects are clearly visible in the *hkl* layers with odd *l*. As Bragg peaks are represented by single pixels on a 721 × 721 grid, they are not easily distinguishable in the presented sections.
